# Predictors of the Treatment Response of Spontaneous Intracranial Hypotension to an Epidural Blood Patch

**DOI:** 10.1097/MD.0000000000003578

**Published:** 2016-05-06

**Authors:** Myong-Hwan Karm, Jae-Hyung Choi, Doohwan Kim, Jun Young Park, Hye Joo Yun, Jeong Hun Suh

**Affiliations:** From the Department of Anesthesiology and Pain Medicine, Asan Medical Center, University of Ulsan College of Medicine, Seoul, Korea.

## Abstract

Spontaneous intracranial hypotension (SIH) is characterized by postural headache because of low cerebrospinal fluid (CSF) pressure. Brain magnetic resonance imaging (MRI) and radioisotope (RI) cisternography can be used to identify the site of a CSF leakage. Although autologous epidural blood patch (EBP) is a very effective treatment modality, some patients require a repeat autologous EBP. We investigated whether autologous EBP responses correlate with surrogate markers of quantitative findings.

All cases of autologous EBP for SIH from January 2006 to December 2014 were enrolled. The demographic variables, number of EBPs, pain scores, RI cisternography (early visualization of bladder activity), and MRI findings (subdural fluid collections, pachymeningeal enhancement, engorgement of venous structures, pituitary hyperemia, and sagging of the brain) were reviewed.

Patients with early bladder activity on RI cisternography had a tendency to need a higher number of autologous EBPs. Only sagging of the brain and no other variables showed a statistically significant negative correlation with the number of autologous EBPs.

The response to autologous EBP may be related to the radiologic findings of early bladder activity on RI cisternography and sagging of the brain on MRI.

## INTRODUCTION

Spontaneous intracranial hypotension (SIH) syndrome can be observed in patients with a postural headache because of low cerebrospinal fluid (CSF) pressure and no previous history of head trauma or dural puncture.^1^ SIH is caused by spontaneous CSF leaks from the spinal meningeal diverticula or the dural rents along nerve sleeves.^2^ SIH is characterized by spontaneous postural headache with nausea, neck stiffness, vomiting, tinnitus, and vertigo in patients with low CSF pressure.^3^ Brain magnetic resonance imaging (MRI) has been used as the diagnostic study of choice for SIH because of its ability to identify characteristic abnormalities such as subdural fluid collections, pachymeningeal enhancement, engorgement of venous structures, pituitary hyperemia, and sagging of the brain.^4,5^ When SIH is suspected on a brain MRI, radioisotope (RI) cisternography, and computed tomographic myelography can be used to identify the site of a CSF leakage.^6–8^ Indirect findings of RI cisternography, such as early visualization of bladder activity, may also be useful in the diagnosis and post-treatment follow-up of CSF leakage.^1^

Although many cases of SIH resolve spontaneously with conservative treatment including bed rest, hydration, caffeine, and analgesics, others require the injection of autologous blood into the epidural space with CSF leakage. Various studies have demonstrated that an autologous epidural blood patch (EBP) is a very effective treatment modality for refractory cases of SIH.^9–13^ Although targeted autologous EBP seems to be effective,^14,15^ some patients require a repeat autologous EBP because of inadequate control of their postural headache.

In our present study, we investigated whether autologous EBP responses correlate with surrogate markers of quantitative findings such as the early visualization of bladder activity in RI cisternography^1^ and subdural fluid collections, pachymeningeal enhancement, engorgement of venous structures, pituitary hyperemia, and sagging of the brain on MRI.^5^ We aimed to identify factors affecting the therapeutic outcomes of autologous EBP in SIH patients in order to help predict the response to autologous EBP.

## METHODS

This retrospective study was approved by the institutional review board of Asan Medical Center (approval number: 2015–0886), and the necessity for obtaining informed consent was waived as we were only reviewing recorded data. All cases of autologous EBP that were performed with the fluoroscopy-guided technique for SIH from January 2006 to December 2014 were reviewed. Patients who met all of the following criteria were included: (1) hospitalized by the neurology department because of symptomatic SIH, (2) received autologous EBP under fluoroscopic guidance, (3) both RI cisternography and brain MRI results were available, and (4) discharged with significant symptom improvement. The exclusion criteria were: (1) incomplete medical records such as an absence of preprocedural and postprocedural pain scores, and (2) absence of formal reports of RI cisternography and brain MRI. Among the 202 patients who underwent autologous EBP at our hospital, only 104 met the inclusion and exclusion criteria.

The following data were collected and analyzed through a review of electronic medical records: demographic variables, number of EBPs, pain scores (VAS; visual analogue scale), early visualization of bladder activity by RI cisternography, and abnormal MRI findings, including subdural fluid collections, pachymeningeal enhancement, engorgement of venous structures, pituitary hyperemia, and sagging of the brain. Early visualization of bladder activity by RI cisternography was defined by the presence of radioactivity in the urinary bladder 1 to 3 hours after a lumbar intrathecal injection of a radioactive tracer.^1^ We evaluated the recorded RI activity in the urinary bladder at 30 minutes and 2 hours after lumbar intrathecal injection of the radioactive tracer for early visualization of bladder activity. MRI data interpretation was based on formal reports of the radiologists.

Patients were diagnosed with SIH if they had at least 2 of the following 3 criteria: orthostatic headache, low CSF pressure, and diffuse pachymeningeal gadolinium enhancement on brain MRI. Orthostatic headache was defined as a headache that occurs or worsens less than 15 minutes after assuming the upright position and disappears or improves less than 30 minutes after resuming the recumbent position. Low CSF pressure was defined as a CSF opening pressure of less than 60 mmH_2_O in the sitting position. Each patient was initially managed with supportive treatments. If these initial supportive treatments failed after 5 to 7 days, patients were referred to the pain clinic and treated with autologous EBP. When patients had orthostatic headache 4 to 5 days after autologous EBP, we performed repeat autologous EBP. The target level of autologous EBP was determined as the level of most increased paraspinal activity on RI cisternography. If additional autologous EBP was required in patients with multiple leakage sites, it was performed at another level that had not been targeted before. Targeted autologous EBPs were performed using a 21-gauge Tuohy needle via a midline or paramedian approach under fluoroscopy C-arm system (OEC 9800, General Electric Healthcare, Little Chalfont, United Kingdom) guidance with the patient in prone position. The epidural space was identified using the loss of resistance technique, and accurate localization was confirmed by ensuring the spread of the injected contrast medium over the targeted epidural space. Thereafter, autologous blood was slowly injected until the patient began to complain of back pain or radicular pain.

### Statistical Analysis

We divided the study subjects into two groups, one including patients in which autologous EBP was performed once (EBP–S), and another comprising cases in which autologous EBP was performed twice or more (EBP–M). Correlation analysis was performed to identify relationships between the number of autologous EBPs and radiologic findings, such as early visualization of bladder activity on RI cisternography, subdural fluid collections, pachymeningeal enhancement, engorgement of venous structures, pituitary hyperemia, and sagging of the brain on MRI. For variables that correlated with the number of autologous EBPs and pre- and post-EBP pain score differences, regression analysis was also conducted to evaluate the causal relationship. These statistical analyses were reviewed by the Medical Statistics Department at our Institute.

## RESULTS

A total of 104 patients who met the inclusion criteria were retrospectively reviewed. There were 39 men and 65 women subjects with a mean age (interquartile range, IR) of 39.2 years (24–66). The mean height (IR) and weight (IR) were 165.4 cm (146–183) and 62.0 kg (43–93). The mean volume (IR) of injected blood was 14.7 mL (9.5–25). The mean pain scores (VAS) before and after the first EBP were 4.6 and 1.1, respectively. The mean number of EBPs (IR) was 2.0 (1–7). Early bladder activity on RI cisternography was evident in 31 (29.8%) patients. Incidences of subdural fluid collections, pachymeningeal enhancement, engorgement of venous structures, pituitary hyperemia, and sagging of the brain on MRI examination occurred in 11 (10.6%), 61 (58.7%), 40 (38.5%), 14 (13.5%), and 22 (21.2%) patients, respectively.

The 104 study patients were divided into two groups, according to the number of autologous EBPs performed, that is, only once (EBP–S, 37 patients), and twice or more (EBP–M, 67 patients). As indicated in Table 1, the demographic characteristics were not significantly different between these two groups. Early bladder activity on RI cisternography showed a statistically significant correlation with the pain score difference between pre- and post-EBP (*P* < 0.05; Spearman rho: −0.240). In addition, patients with early bladder activity on RI cisternography had a tendency to need a higher number of autologous EBPs (Fisher exact test, *P* = 0.03) (Table 2). By correlation analysis, only sagging of the brain and no other variables showed a statistically significant negative correlation with the number of autologous EBPs (*P* < 0.01; Spearman rho: −0.259).

**TABLE 1:**
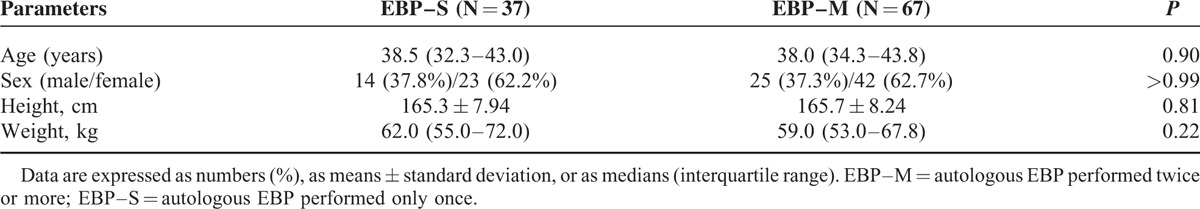
Characteristics of Spontaneous Intracranial Hypotension Patients Who Receive Single or Multiple Autologous Epidural Blood Patch Treatments

**TABLE 2:**
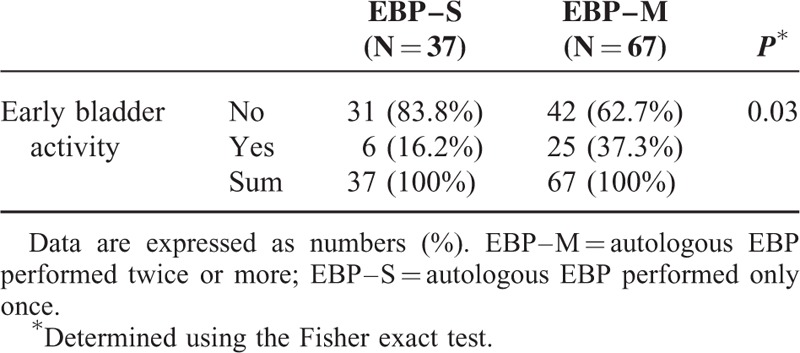
Proportions of the Study Patients With Early Bladder Activity

## DISCUSSION

Scahltenbrand initially introduced “aliquorrhea” in 1938 to describe a patient with SIH,^16^ and also suggested that there were 3 possible causes of SIH syndrome: increased CSF absorption, reduced CSF production, and CSF leakage.^1^ CSF leakage into the spinal epidural space causes several symptoms of SIH such as spontaneous orthostatic headache with nausea, neck stiffness, vomiting, tinnitus, and vertigo.^3^ Orthostatic headache due to SIH syndrome usually resolves spontaneously, but autologous EBP should be considered to relieve symptoms in refractory cases.^17^ Autologous EBP has been recommended as the treatment of choice in patients who have failed initial noninvasive treatments such as hydration and bed rest, a generous caffeine intake, and the use of an abdominal binder.^18,19^ The effect of autologous EBP is 2-fold: (1) an immediate effect related simply to volume replacement by compressing the dura mater; and (2) a subsequent latent effect related to sealing of the leakage.^20^ In SIH syndrome, the success rate with a first autologous EBP has been reported to be 30 to 87%.^14,18^ Many patients, however, require more than one autologous EBP or other treatments. We have experienced cases in which 7 autologous EBPs were needed to achieve lasting relief. Hence, we decided to investigate the unresponsiveness to autologous EBP in such patients.

We had aimed in a previous study to elucidate the relationship between autologous EBP responses and RI cisternographic findings, hypothesizing that the number of autologous EBPs would be increased if the number of CSF leakage levels on RI cisternography were increased. In our previous study, we found that the response to autologous EBP was related to the severity of symptoms but not to the number and location of CSF leakages.^21^ One of the limitations of our previous study was that we only dealt with the number of leakage sites without any quantitative analysis of the amount of leakage because there are no proper tools to measure this.^21^ We, therefore, decided to use 6 surrogate markers of the quantitative evaluation of leakage based on previous studies.^1,2,22^ The first marker was derived from the study of Morioka et al^1^ who evaluated four indirect findings of CSF leakage on RI cisternography, such as the early visualization of bladder activity, no visualization of activity over the brain convexities, rapid disappearance of spinal activity, and abnormal visualization of the root sleeves. These authors demonstrated early visualization of bladder activity in RI cisternography in all patients with CSF leakage.^1^ As the release of radioactive tracer into the systemic circulation by CSF leakage increases, early bladder activity can be considered as a surrogate marker of CSF leakage. Severe intracranial hypotension is characterized by 5 MRI findings described by Schievink, which we used as the additional surrogate markers: (1) subdural fluid collections or hygromas, (2) pachymeningeal enhancement, (3) engorgement of venous structures, (4) pituitary hyperemia, and (5) sagging of the brain.^2,22^ We investigated in our current study whether autologous EBP responses correlate with these 6 surrogate markers of the quantitative levels of CSF leakage.

Early bladder activity on RI cisternography showed a significant correlation with the pain score and with the number of autologous EBPs in our current patient series. Although there was no significant difference between these patients and the cases without early bladder activity on RI cisternography, multiple autologous EBP was often needed for patient with early bladder activity on RI cisternography. Therefore, we suggest from our current findings that early bladder activity on RI cisternography is a predictive factor for the autologous EBP response.

Among the 5 MRI surrogate markers we evaluated, only brain sagging showed a significant correlation with autologous EBP responses. Although this was not significantly different in the cases without sagging of the brain on MRI, the patients with sagging of the brain showed better autologous EBP responses. Sagging was defined as a downward displacement of the brain because of CSF leakage. The better responses to autologous EBP treatments in patients with sagging of the brain were considered dependent on the fact that it is helpful to raises the CSF pressure by preventing CSF leakage sites. Thus, we identified early bladder activity on RI cisternography and sagging of the brain as factors affecting the responsiveness to autologous EBP. These variables may, therefore, give some useful predictive information on the therapeutic effects of autologous EBP.

Our study had several limitations of note. First, we analyzed early visualization of bladder activity by RI cisternography because we could not analyze the actual RI activity. If we could get the raw data and analyze the actual RI activity in RI cisternography, this would give more reliable results. Second, we considered the therapeutic effect of autologous EBP to be poor if the number of autologous EBPs increased. However, the number of autologous EBPs is not equivalent to the therapeutic effects of this treatment. This may introduce an error; however, the patients were discharged after the symptoms of SIH syndrome improved. If a postural headache worsened with ambulation or in a sitting position, autologous EBP was conducted again until the symptoms of SIH improved. Therefore, we decided to determine the treatment response by using the number of autologous EBPs, and this modality of evaluation should have less influence on the results.

In conclusion, autologous EBP is an effective treatment method for managing SIH, and the response to it may be related to the radiologic findings of early bladder activity on RI cisternography and sagging of the brain on MRI. However, the number of autologous EBPs necessary to achieve symptomatic relief does not correlate with other MRI abnormal findings, such as subdural fluid collections, pachymeningeal enhancement, engorgement of venous structures, and pituitary hyperemia. These results are meaningful because they reveal that the therapeutic effect of EBP on SIH can be predicted.
